# Estimation of Anti-SARS-CoV-2 IgM/IgG Seroprevalence Among Non-Vaccinated and Vaccinated University Students: A Cross-Sectional Egyptian Study

**DOI:** 10.3390/v17030378

**Published:** 2025-03-06

**Authors:** Ahmed E. Taha, Ibrahim Amer, Shimaa El Sharawy, Amany A. Ghazy

**Affiliations:** 1 Microbiology and Immunology Unit, Department of Pathology, College of Medicine, Jouf University, Sakaka 72388, Saudi Arabia; aeattia@ju.edu.sa; 2 Medical Microbiology and Immunology Department, Faculty of Medicine, Mansoura University, Mansoura 35516, Egypt; 3 Department of Hepatology, Gastroenterology and Infectious Diseases, Faculty of Medicine, Kafr Elsheikh University, Kafr Elsheikh 33516, Egypt; ebrahiem_amier@med.kfs.edu.eg; 4 Department of Tropical Medicine and Infectious Diseases, Faculty of Medicine, Tanta University, Tanta 31527, Egypt; shaimaa.elsharawy@med.tanta.edu.eg

**Keywords:** COVID-19, healthy, immunization, SARS-CoV-2, serological testing, vaccines, viruses

## Abstract

It is essential to comprehend the humoral immune response to severe acute respiratory syndrome coronavirus-2 (SARS-CoV-2) and its vaccines to maximize the effectiveness of anti-SARSCoV-2 community immunization efforts. The aim of this cross-sectional study was to determine the seroprevalence of anti-SARS-CoV-2 IgM/IgG among newcomer students at Kafr Elsheikh University in Egypt, whether they had been vaccinated or not. Blood samples from 400 healthy newcomer students (200 non-vaccinated and 200 vaccinated) were evaluated for the presence of anti-SARS-CoV-2 IgM/IgG using colloidal gold immunochromatography lateral flow immunoassay cards, and then the results were confirmed by using specific ELISA tests. The prevalence of anti-SARS-CoV-2 antibodies among the participants (*n* = 400) was 56.3% for IgG and 13.3% for IgM. Regarding the non-vaccinated participants, 55.0% were females, the mean age was 18.2 years, and the mean BMI was 25.35. Regarding the vaccinated participants, 58.5% were females, the mean age was 18.1 years, and the mean BMI was 25.3. There were statistically non-significant correlations (*p* ˃ 0.05) between gender, BMI, and each of IgM- or IgG-positivity in both vaccinated and non-vaccinated groups. In total, 41.5% and 48.5% of the anti-SARS-CoV-2 IgM-positive and anti-SARS-CoV-2 IgG-positive participants were non-vaccinated, respectively. Furthermore, 58.5% and 51.5% of the anti-SARS-CoV-2 IgM-positive and anti-SARS-CoV-2 IgG-positive participants were vaccinated, respectively. No statistically significant association (*p* ˃ 0.05) in immunoglobulins positivity between the anti-SARS-CoV-2 non-vaccinated, and vaccinated groups. The anti-SARS-CoV-2 immunological response of nonsmokers, people who exercise regularly, and those who take vitamin supplements, eat a balanced diet, and use certain herbs is noteworthy. Among the vaccinated subjects, 96.6%, 25.0%, 31.9%, 45.7%, and 7.8% of the IgG-positive group, versus 97.2%, 60.6%, 64.2%, 52.3%, and 6.4% of the IgG-positive non-vaccinated group, were nonsmokers, exercisers, and those taking vitamin supplements, eating a balanced diet, and using herbs, respectively. Furthermore, 93.5%, 32.3%, 35.5%, 48.4%, and 6.5% of the IgM-positive vaccinated group, versus 100.0%, 63.6%, 81.8%, 45.5%, and 4.5% of the IgM-positive non-vaccinated participants, were nonsmokers, physical exercisers, vitamin supplement users, balanced eaters, and herbalists, respectively. Persons who are free from comorbidities, young, non-obese, non-smokers, engage in physical exercise, take vitamins, eat a balanced diet, and use certain immunostimulant herbal supplements, all have a strong anti-SARS-CoV-2 humoral immune response, even if they were not vaccinated. During pandemics, vaccination of this group should not be a priority to preserve vaccine doses for high-risk vulnerable people. Even if there is a lockdown during an anticipated future epidemic or pandemic, we should prioritize healthy eating and lifestyle choices, along with increasing physical activity.

## 1. Introduction

Coronaviruses belong to the Coronaviridae family and are among the largest enveloped, positive-sense, single-stranded RNA viruses, with a genomic size of 30 Kb [1]. The severe acute respiratory syndrome coronavirus-2 (SARS-CoV-2) appears to have originated in bats and transferred to animals in wildlife markets, which served as the virus’s intermediate hosts before its transmission to humans, causing the coronavirus disease-2019 (COVID-19) [2].

Four conserved structural proteins are encoded by the SARS-CoV-2 genome: spike (S), envelope (E), membrane (M), and nucleocapsid (N). The S protein, which is made up of transmembrane, cytoplasmic S1 and S2 subunits, starts SARS-CoV-2 infection in the host cells. The N-terminal domain (NTD), receptor-binding domain (RBD), subdomain 1 (SD1), and subdomain 2 (SD2) are additional divisions of the S1 subunit. The RBD in the SARS-CoV-2 S1 subunit promotes virus entrance by attaching to the host cell’s cellular (angiotensin-converting enzyme 2; ACE2) receptor. The body’s humoral and cellular immunity are activated when the virus enters the cell. To combat COVID-19, the humoral immune response is very essential [3].

SARS-CoV-2 clinical pictures range from asymptomatic carriage to acute respiratory distress (ARDS) and deadly pneumonia. Asymptomatic people can transfer infectious viruses via their respiratory secretions. Elderly patients with underlying comorbidities frequently have a severe clinical picture. More research is needed to fully understand the full pathogenesis of this lethal viral disease [4,5].

The recognition of viral antigens by B cell receptors or other antigen-presenting cells induces the anti-SARS-CoV-2 immunoglobulins (Igs) formation. As a result, B cells and T cells that target SARS-CoV-2 are activated and proliferate. B cells release a variety of anti-bodies, including IgM, IgG, and IgA, which can destroy the virus, help other immune cells remove it, stop viral cell invasion, or spike protein-mediated cell fusion [6]. Most symptomatic cases and asymptomatic carriers can induce specific anti-SARS-CoV-2 humoral immune responses, with the primary phase (IgM) showing up 3–10 days after infection and the secondary phase (IgG) following two weeks later, lasting for months [7,8].

Anti-SARS-CoV-2 vaccine development was accelerated by the COVID-19 pandemic [9]. The anti-SARS-CoV-2 vaccines have proven to be a successful immunization strategy for preventing COVID-19. To stop COVID-19 from worsening, vaccines create a particular long-term immunological memory against the virus [10].

There are many COVID-19 vaccine technologies from different manufacturers. Examples of mRNA technology include the BNT162b2 and mRNA-1273 vaccines produced by Pfizer/BioNTech and Moderna manufacturers, respectively, [11,12]. The ChAdOX1/AZD1222, Ad26.COV2, and Ad5.nCOV vaccines are examples of viral vector technology introduced by AstraZeneca/Oxford, Johnson & Johnson, and Casino Biologics manufacturers, respectively, [13,14]. Sinovac, Sinopharm, and Bharat Biotech manufacturers depended on inactivated virus technology for developing the Corona Vac, BBIBP-CorV/WIBP-CorV, and BBV-152/Covaxin vaccines, respectively, [15,16,17].

As of 15 December 2024, there were 777,074,803 confirmed COVID-19 cases worldwide, including 7,079,142 deaths, according to the WHO. As of 31 December 2023, 13.64 billion doses of anti-SARS-CoV-2 vaccines had been distributed all over the world, with 67% of the population having received the entire primary vaccination series and 32% having received at least one booster dose [18]. As of 15 December 2024, the WHO received reports of 516,023 confirmed cases of COVID-19 in Egypt, including 24,830 deaths. As of 31 December 2023, 102.19 million doses of anti-SARS-CoV-2 vaccine had been given, with 41% of the population having finished the primary vaccination series and 15% having received at least one booster dose in Egypt [19].

For SARS-CoV-2 control program to be successful, early testing techniques are essential. The gold standard for diagnosing individuals who are currently infected is the use of RT-PCR to detect SARS-CoV-2-RNA. SARS-CoV-2-exposed persons generate antibodies can be identified by serological testing. Thus, extensive serological testing might be utilized to represent the true spread of SARS-CoV-2 in the community. The most widely used serological techniques to identify anti-SARS-CoV-2 immunoglobulins are enzyme-linked immunosorbent assay (ELISA), lateral flow immunoassay (LFIA), and chemiluminescence immunoassay (CLIA). The typical specificity and sensitivity of LFIA cards depends on the manufacturer [20].

People who recover from COVID-19 have strong and long-lasting adaptive immunity to SARS-CoV-2, including memory B cells, memory T cells, and neutralizing Igs. Both the original and variant strains of SARS-CoV-2 can be recognized by these immune components, albeit the effectiveness of this protection may change based on how different the original and variant strains are from one another. Furthermore, vaccination can boost adaptive immune responses against SARS-CoV-2 by producing larger quantities of T cells and antibodies than a natural infection. Additionally, vaccination lowers the risk of serious illness and reinfection with SARS-CoV-2 variants. It is well established that adaptive immunity to SARS-CoV-2 is a complicated phenomenon that differs between people and populations depending on many parameters, including genetic background, age, comorbidities, immunosuppression, auto-immunity, immunodeficiency, early innate immunity, exposure history to SARS-CoV-2 or other coronaviruses, and viral load. For significant protection, those with compromised immune systems typically need more frequent or more effective immunization regimens. In addition to these variables, it has been demonstrated that the type of exposure—infection (naturally acquired immunity), vaccination with one or more vaccine types (vaccine induced immunity), or infection followed by vaccination or vice versa (hybrid immunization)—affects the quality and duration of the acquired immunity [21].

Since its emergence, SARS-CoV-2 information has rapidly increased, although many unanswered questions still remain. The kinetics of antibody production during SARS-CoV-2 infection are influenced by the various patient variables [22]. Since healthcare workers (HCWs) were given priority in vaccination campaigns, post-vaccination antibody response has primarily been reported in these individuals [23]. Nevertheless, little is now known about the immune response’s variability and how it varies among specific individuals. Optimizing community immunization programs against SARSCoV-2 requires an understanding of the humoral immune response to SARSCoV-2 and its vaccines. Data regarding the humoral immunity of vaccinated and unvaccinated individuals for SARS-CoV-2 in Kafr Elsheikh city, Egypt, are lacking. We aimed to assess the seroprevalence of anti-SARS-CoV-2 IgM/IgG among newcomer students in Kafr Elsheikh University, Egypt, compare the results of non-vaccinated group with a cross matched vaccinated group, and correlate the results to participants’ anthropometrics, relevant COVID-19 history, and lifestyle.

## 2. Methodology

### 2.1. Design of the Study

During the four months from July to October 2022, this cross-sectional study was carried out on newcomer students who consented to participate at Kafr Elsheikh University in Egypt. From the 16,000 newcomer students at Kafr Elsheikh University, the sample size was determined using the online (Roasoft) sample size calculator (http://www.raosoft.com/samplesize.html, accessed on 16 May 2022) with a 50% response distribution, a 95% confidence level, and a 5% margin of error. In total, 400 healthy participants were involved in the study (200 participants were non-vaccinated, and the other 200 participants were vaccinated).

Exclusion criteria included newcomer students in Kafr Elsheikh University with comorbidities (diabetes, hypertension, cancer, chronic respiratory, chronic inflammatory, autoimmune, kidney, heart and liver diseases, bleeding/coagulation disorders, etc.), and those who are currently receiving medications (anti-diabetic, antihypertensive, anti-cancer, anti-rheumatoid, immunosuppressive drugs, blood thinners, etc.).

Inclusion criteria included newcomer students in Kafr Elsheikh University who agreed to participate in the study, free from comorbidities, and who are currently not receiving medications. At their initial appearance, the students were willingly recruited one after the other until the necessary sample size was reached. After receiving information about the study, each participant was asked to sign the informed consent formula. After gathering the relevant data using a data collection template, a blood sample was taken for analysis.

### 2.2. Collection of Data

The collected data include gender, age, body mass index (BMI; calculated as weight in kg/height in m^2^), anti-SARS-CoV-2 vaccination history, lifestyle (smoking, physical activity, vitamin supplements, healthy diet, and herbal supplements), previous COVID-19-like symptoms, previous hospital isolation due to COVID-19-like symptoms, previous RT-PCR-positive SARS-CoV-2 diagnosis, previous RT-PCR-negative SARS-CoV-2 test, prior interaction with an individual who had a positive SARS-CoV-2-RNA PCR test, and prior interaction with an individual exhibiting COVID-19-like symptoms who either had negative RT-PCR results or no RT-PCR testing.

### 2.3. Collection of Blood Samples

Following skin antisepsis using an alcohol-based swab, a 5-milliliter sample of blood was taken for analysis. A disposable micropipette was used to transfer a tiny drop of blood into the LFIA card, and the remaining amount of blood (with anticoagulant EDTA) was used to confirm the results by using a specific ELISA test. For appropriate disposal, bio-wastes were gathered in sharp boxes and yellow waste bags.

### 2.4. Immunological Detection of Antibodies by LFIA Cards

Using colloidal gold immuno-chromatography LFIA cards (SunLong Biotech Co., Ltd., Hangzhou, Zhejiang, China) with high sensitivity (93% for IgM, and 94% for IgG) and specificity (94% for IgM, and 96% for IgG) values, anti-SARS-CoV-2 IgM and IgG were initially tested in a small blood drop, as described before [20,24].

### 2.5. Immunological Detection of Antibodies by Specific ELISA Test

When the samples arrived at the laboratory, they were processed aseptically to prevent contamination. Blood samples were centrifuged for 10 min at 3000 rpm after being left at room temperature for 10 to 20 min. After being separated, the plasma supernatant was stored at −80 °C till the time of ELISA testing. Using sensitive (>95%) and specific (>95%) ELISA kits, anti-SARS-CoV-2 antibodies (IgM and IgG) were immunologically tested according to the manufacturers’ instructions (catalog number SL3089Hu for anti-SARS-CoV-2 IgM, catalog number SL0521Hu for anti-SARS-CoV-2 IgG, SunLong Biotech Co., Ltd., Hangzhou, Zhejiang, China) at 450 nm using the ELISA reader (Robonik, Mumbai, India). Every run contained blank, negative, and positive controls. The cut-off value was set at OD ≥ 0.25. The concentration of immunoglobulins in the samples was directly correlated with the color intensity [25].

### 2.6. Analysis of the Data

Data were entered into the computer and processed with IBM SPSS software version 20.0. (Armonk, NY, USA: IBM Corporation). Categorical data were presented as numbers and percentages. The chi-square test was used to compare the two groups. Alternatively, the Fisher exact correction/Monte Carlo correction test was used when more than 20% of the cells had an expected count of less than 5. The Kolmogorov–Smirnov test was used to assess the normality of continuous data. The mean, standard deviation, median, and range (minimum and maximum) were used to express the quantitative data. Two sets of quantitative data that were regularly distributed were compared using the Student *t*-test. On the other hand, two sets of quantitative data that were not regularly distributed were compared using the Mann–Whitney test. At the 5% level, the gathered results’ significance was determined.

## 3. Results

In total, 400 healthy participants (200 vaccinated, and 200 non-vaccinated against SARS-CoV-2) were tested for anti-SARS-CoV-2 antibodies by LFIA cards, then the results were confirmed by using specific commercially available indirect ELISA tests. Regarding the vaccinated participants (*n* = 200; 50.0%), 58.5% were females, 41.5%% were males, the mean age was 18.1 years (±0.3 SD), and the mean BMI was 25.3 (±2.8 SD). Regarding the non-vaccinated participants (*n* = 200; 50.0%), 55.0% were females, 45.0%% were males, the mean age was 18.2 years (±0.4 SD), and the mean BMI was 25.35 (±2.58 SD).

The prevalence of anti-SARS-CoV-2 antibodies among the participants (*n* = 400) was 56.3% (*n* = 225) for IgG and 13.3% (*n* = 53) for IgM. Seropositivity for both anti-SARS-CoV-2 IgG and IgM was 7.3% (*n* = 29). In total, 41.5% (*n* = 22/53) and 48.5% (*n* = 109/225) of anti-SARS-CoV-2 IgM-positive and anti-SARS-CoV-2 IgG-positive participants were non-vaccinated, respectively. Furthermore, 58.5% (*n* = 31/53) and 51.5% (*n* = 116/225) of anti-SARS-CoV-2 IgM-positive and anti-SARS-CoV-2 IgG-positive participants were vaccinated, respectively. There was no statistically significant association (*p* > 0.05) in immunoglobulins positivity between the anti-SARS-CoV-2 non-vaccinated and vaccinated groups, as shown in Table 1.

It is important to note that the majority of individuals who received anti-SARS-CoV-2 vaccinations at the time of the study received Sinopharm/Sinovac-CoronaVac (*n* = 121; 60.5%) followed by AstraZeneca (*n* = 51; 25.5%), and Pfizer (*n* = 28; 14.0%) vaccines, as shown in Table 2. Most of the vaccinated participants received two anti-SARS-CoV-2 vaccination doses (*n* = 192; 96.0%) at the time of the study. Totally, 54.7% (*n* = 29/53) and 48.0% (*n* = 108/225) of anti-SARS-CoV-2 IgM-positive and anti-SARS-CoV-2 IgG-positive participants received two anti-SARS-CoV-2 vaccination doses, respectively. There was a statistically significant association (*p* = 0.029 *) between receiving two doses of the vaccines and anti-SARS-CoV-2 IgG positivity. The non-vaccinated participants expressed nearly equal levels of anti-SARS-CoV-2 IgM (*n* = 22/53; 41.5%) and anti-SARS-CoV-2 IgG (*n* = 109/225; 48.5%) positivity compared to participants who received two doses of anti-SARS-CoV-2 vaccines, as shown in Table 3.

Moreover, Table 4 and Table 5 present a comparison of the anthropometrics, COVID-19 history, and lifestyle of 200 vaccinated and 200 non-vaccinated participants, respectively, who were stratified according to the positivity or negativity of anti-SARS-CoV-2 IgM and IgG in their blood. There were statistically non-significant correlations between gender, BMI, and each of IgM- or IgG-positivity in both vaccinated and non-vaccinated groups.

There were statistically significant correlations between being the age of 18.1 years, previous experience of COVID-19-like symptoms on one hand, and the anti-SARS-CoV-2 IgG positivity among vaccinated participants on the other hand, with (*p* = 0.025 *) and (*p* < 0.001 *), respectively, as shown in Table 4.

There were statistically significant correlations between previous experience of COVID-19-like symptoms, not receiving herbal supplements on one hand, and anti-SARS-CoV-2 IgG positivity among non-vaccinated participants on the other hand, with (*p* < 0.001 *) and (*p* < 0.001 *), respectively, as shown in Table 5.

All the participants (*n* = 400) had never performed SARS-CoV-2-RNA testing by RT-PCR before serology testing in the conducted study, and none of them had ever been hospitalized due to COVID-19-like symptoms.

Even though most participants (58.0%; *n* = 232/400) had previously had symptoms similar to COVID-19, 86.6% (*n* = 201/232) and 27.2% (*n* = 63/232) of them were negative for anti-SARS-CoV-2 IgM and anti-SARS-CoV-2 IgG, respectively. Participants with a history of COVID-19-like symptoms were more likely to have anti-SARS-CoV-2 IgM seronegativity and anti-SARS-CoV-2 IgG seropositivity. However, 42.0% (*n* = 168/400) of the people who participated had never experienced COVID-19-like symptoms before, and 13.1% (*n* = 22/168) and 33.3% (*n* = 56/168) of them had anti-SARS-CoV-2 IgM-positive and IgG-positive results, respectively. Participants who had never experienced COVID-19-like symptoms before were more likely to have anti-SARS-CoV-2 IgM and IgG seronegativity.

Most of the participants were anti-SARS-CoV-2 IgM-negative and IgG-negative, with prevalence of 83.5% (*n* = 71/85) and 54.1% (*n* = 46/85), respectively, even though 21.3% (*n* = 85/400) were exposed to someone who was SARS-CoV-2 RT-PCR-positive. In addition, most participants (62.5%; *n* = 250/400) reported having come across an individual exhibiting symptoms like COVID-19 but lacking RT-PCR testing or having a negative RT-PCR test for SARS-CoV-2-RNA. Of these, 85.6% (*n* = 214/250) were IgM-negative and 58.0% (*n* = 145/250), were anti-SARS-CoV-2 IgG-positive.

The anti-SARS-CoV-2 immune response of non-smokers, persons practicing physical activity, and those taking vitamin supplements, having a healthy diet, and using some herbs is striking. Among the vaccinated participants, 96.6% (*n* = 112/116), 25.0% (*n* = 29/116), 31.9% (*n* = 37/116), 45.7% (*n* = 53/116), and 7.8% (*n* = 9/116) of the IgG-positive group were nonsmokers, participants who exercise, take vitamin supplements, have a balanced diet, and use certain herbs, respectively. Additionally, 93.5% (*n* = 29/31), 32.3% (*n* = 10/31), 35.5% (*n* = 11/31), 48.4% (*n* = 15/31), and 6.5% (*n* = 2/31) of the IgM-positive group were non-smokers, participants who engage in physical exercise, those who take vitamin supplements, eat a balanced diet, and use certain herbs, respectively, as shown in Table 4.

Likewise, among the non-vaccinated participants, 97.2% (*n* = 106/109), 60.6% (*n* = 66/109), 64.2% (*n* = 70/109), 52.3% (*n* = 57/109), and 6.4% (*n* = 7/109) of the IgG-positive group were those who do not smoke, engage in physical activity, take vitamin supplements, eat a nutritious diet, and use herbal supplements, respectively. Moreover, 100.0% (*n* = 22/22), 63.6% (*n* = 14/22), 81.8% (*n* = 18/22), 45.5% (*n* = 10/22), and 4.5% (*n* = 1/22) of the IgM-positive group were non-smokers, persons practicing physical activity, taking vitamin supplements, on a healthy diet, and using some herbs, respectively, as shown in Table 5.

## 4. Discussion

To prevent emerging viruses from reaching the pandemic stage and having catastrophic consequences on the world’s population and economy, unconventional strategies necessitate political will, global vision, and cooperation between pharmaceutical companies, governments, and the World Health Organization (WHO). The lessons learnt from dealing with the SARS-CoV-2 pandemic can offer an important insight into how to best respond to future attacks of this nature. In this prospective study, we aimed to assess the seroprevalence of IgM/IgG against SARS-CoV-2 among newcomer students from Kafr Elsheikh University, Egypt, compare the results of non-vaccinated group with a cross matched vaccinated group, and correlate the results to participants’ anthropometrics, relevant COVID-19 history, and lifestyle.

The anti-SARS-CoV-2 antibodies’ prevalence among the conducted study’s the participants was 56.3% for IgG and 13.3% for IgM. The seroprevalence of IgG/IgM against SARS-CoV-2 is variable worldwide. Lower seroprevalence of IgG and higher seroprevalence of IgM were detected in Saudi Arabia among vaccinated/unvaccinated participants (42.3%, and 45.8%, respectively) [24]. Another study from Saudi Arabia reported very low seroprevalence of IgG against SARS-CoV-2 (9%), whereas the seroprevalence of IgM against SARS-CoV-2 was higher (65%) among unvaccinated participants [25]. In Iraq, the seroprevalence of IgG and IgM against SARS-CoV-2 was 26.58% and 31.08%, respectively, [26], and this was also measured in Iran (33% and 22%, respectively) [27], in Sweden (6.8% and 1.7%, respectively) [28], and in the USA (4.16% and 2.49%, respectively) [29]. The great variability in seroprevalence of IgG/IgM against SARS-CoV-2 among studies may be due to a variety of parameters, including the tested population, genetic background, age, history of exposure to SARS-CoV-2 or different coronaviruses, viral load, history of anti-SARS-CoV-2 vaccination, comorbidities, and drug intake.

In the current study, the included participants were free from comorbidities, and they were not receiving medications. Comorbidities impair the host immune response against SARSCoV-2. According to numerous studies, unvaccinated and vaccinated individuals who have comorbidities are more likely than those who do not to have COVID-19 infection, severe complications, and require recurrent hospitalizations. Thus, even if they have been vaccinated, people with common comorbidities should exercise caution [30,31,32]. Hospitalized COVID-19 patients with comorbidities are treated with more sophisticated medications that may increase the likelihood of potential drug–drug interactions [33], even with the recently approved anti-SARSCoV-2 drugs [34]. The seroconversion is lower in immunocompromised individuals, due to medical conditions or disease treatments, than in healthy individuals [3].

In the performed study, regarding the vaccinated participants, 58.5% were females, 41.5% were males, the mean age was 18.1 years (±0.3 SD), and the mean BMI was 25.3 kg/m^2^ (±2.8 SD). Regarding the non-vaccinated participants, 55.0% were females, 45.0% were males, the mean age was 18.2 years (±0.4 SD), and the mean BMI was 25.35 kg/m^2^ (±2.58 SD). There were statistically non-significant correlations between gender, BMI, and each of IgM or IgG positivity in both vaccinated and non-vaccinated groups. There were statistically significant correlations between being the age of 18.1 years on one hand, and anti-SARS-CoV-2 IgG positivity among vaccinated participants on the other hand, with (*p* = 0.025 *) and (*p* < 0.001 *), respectively, as shown in Table 4.

The relation between gender and age differences and the vaccine-induced humoral immune response gained a great attention. Generally, post-vaccination antibody responses are often larger in females than in males, and immune senescence reduces vaccine responses in older adults [3]. Following the first dose (1D) of CoronaVac, it was found that seropositivity was higher in females (84.6%) compared to males (70.6%) [35]. Furthermore, following 1D of CoronaVac, it was reported that females had higher seropositivity (90.5%) than males (79.4%). Additionally, females had higher antibody levels. After 1D, the median IgG level was 761.6 AU/mL for females and 626.6 AU/mL for males; following the second dosage (2D), these levels rose to 1252 and 959.6 AU/mL, respectively, [36]. Furthermore, the IgG titer of females (263.6 ± 158.0 U/mL) was considerably greater than that of males (209.9 ± 137.6 U/mL) following 2D of BNT162b2 [37]. The gender differences in BBV-152 (COVAXIN) and Covishield vaccinations were also noted by Choudhary and his research team, although they were not statistically significant [17]. Moreover, women’s titer increases by 2.19 times between 1D and 2D of the AZD1222 vaccine, whereas men’s increases by 1.03 times [38]. Hormonal variations can account for sex-based variations in the immunological response. Numerous immune cells, including B lymphocytes, have been found to have estrogen receptors that are controlled by estrogen levels [39,40]. Indeed, it has been demonstrated that testosterone can prevent the formation of immunoglobins, whilst estrogen can increase it [41].

Khoury and his colleagues observed that those aged > 50 years who received the BNT162b2 vaccination had lower antibody titers than patients under 30 years: mean titer was 14,786 ± 15,471 AU/mL and 33,660 ± 20,771 AU/mL, respectively, [42]. According to Mishra et al., at all time points assessed, those under 45 had a greater mean titer than those over 45 [38]. Following 1D of CoronaVac, researchers observed seropositivity of 85.4%, 68.2%, and 37.5% among HCWs aged 18–34, 35–59, and ≥60 years, respectively, [35]. However, following 2D, no clear associations between age and antibody concentrations were found [16]. Additionally, among AZD1222 recipients, investigators found no statistically significant variation in post-vaccination antibody production and persistence by age or gender [17].

The mean BMI for the participants (25.3 kg/m^2^) in the current study denotes that they are not obese (obesity is at BMI ≥ 30 kg/m^2^ at the age around 19 years according to the WHO [43]). Obesity was found to be substantially linked with prolonged COVID syndrome and post-COVID-19 condition (PCC). Obesity and PCC both have metabolic pro-inflammatory states that encourage inflammatory processes and the long-lasting signs and symptoms that go along with them [31]. Adipose tissue is a source of inflammatory cytokines and adipokines that regulate glucose and insulin resistance, as well as high ACE2 levels which may indicate a tendency for SARS-CoV-2. Excess adipose tissue may hinder the immune cells’ ability to obtain nutrients [44]. Adipocyte hypertrophy brought on by obesity results in low levels of inflammation and insulin resistance [45]. Furthermore, T cell dysfunction and compromised immunological responses are caused by the hyperleptinemia and hyperinsulinemia that accompany obesity [46]. Following vaccination, these immune cell suppression ways may decrease the production of antibodies [32]. A possible early decline of vaccine-induced antibodies associated with obesity is also suggested by some research that reported earlier waning of vaccine-induced antibodies in obese people than in people of normal weight [47,48]. When a person contracts COVID-19 after completing their original immunization sequence, this is referred to as a breakthrough infection. Those who require one or more booster doses of vaccination do so primarily because of the waning of vaccine-induced antibodies and vaccine variant mismatch [30].

It is vital to notice that most persons who received anti-SARS-CoV-2 vaccinations at the time of the study received Sinopharm/Sinovac-CoronaVac (60.5%) followed by AstraZeneca (25.5%), and Pfizer (14.0%) vaccines, as shown in Table 2. The anti-SARS-CoV-2 IgG positivity and receiving two doses of the vaccinations were significantly correlated (*p* = 0.029 *). The Sinopharm/Sinovac-COVID-19 vaccines (henceforth referred to as Chinese COVID-19 vaccines) are inactivated vaccines. All adults without contraindications between the ages of 18 and 59 were eligible to receive these vaccinations [49]. Because inactivated vaccines are simpler to produce in underdeveloped nations and have fewer side effects, they may result in greater economic advantages by reducing work absences; thus, they were preferred in Egypt.

A study conducted in Egypt found that the BBIBP-CorV vaccination is associated with an infection protection and immune response like ChAdOx1 nCoV-1 vaccine. It’s interesting to note that there was no discernible difference in the levels of anti-S1 and anti-RBD antibodies between the two immunization groups [50]. The Egyptian Drug Authority has certified Sinopharm’s Chinese vaccinations as the first primary vaccines [51]. Egypt is now the largest vaccine manufacturer in the Middle East and Africa, producing one billion doses of China’s Sinovac vaccine annually [52]. Another Egyptian study found that the AstraZeneca vaccine had more adverse effects than the Sinopharm vaccine, but the Sinopharm vaccine had a considerably greater COVID-19 infection rate following vaccination. Individuals who have received the Sinopharm vaccine should have a booster shot, because they have a much greater risk of contracting COVID-19 following vaccination [53]. Furthermore, the Egyptian government prioritized vaccination for healthcare personnels and elderly people with chronic illnesses [54].

In total, 41.5% of participants who tested positive for anti-SARS-CoV-2 IgM and 48.5% of those who tested positive for anti-SARS-CoV-2 IgG were not vaccinated. Additionally, 58.5% of participants who tested positive for SARS-CoV-2 IgM and 51.5% of those who tested positive for SARS-CoV-2 IgG received vaccinations. No statistically significant association (*p* ˃ 0.05) was found in immunoglobulins positivity between the anti-SARS-CoV-2 non-vaccinated and vaccinated groups, as shown in Table 1. Furthermore, non-vaccinated participants had virtually identical levels of anti-SARS-CoV-2 IgM (22/53; 41.5%) and anti-SARS-CoV-2 IgG (109/225; 48.5%) positivity compared to participants who received two doses of anti-SARS-CoV-2 vaccines (29/53; 54.7% for IgM, and 108/225; 48.0% for IgG, respectively), as shown in Table 3. This could indicate the adequate anti-SARS-CoV-2 immune response of the examined healthy participants, whether or not they were vaccinated.

Previous infection with SARS-CoV-2 is another issue that require attention. There were statistically significant correlations between having previous experience of COVID-19-like symptoms on one hand, and the anti-SARS-CoV-2 IgG positivity among vaccinated participants on the other hand, with (*p* = 0.025 *) and (*p* < 0.001 *), respectively, as shown in Table 4. There were statistically significant correlations between previous experience of COVID-19-like symptoms on one hand, and the anti-SARS-CoV-2 IgG positivity among non-vaccinated participants on the other hand, with (*p* < 0.001 *) and (*p* < 0.001 *), respectively, as shown in Table 5

When compared to previously uninfected individuals, numerous studies demonstrated the benefits of vaccination in previously infected individuals, as evidenced by higher antibody levels within a shorter time frame following the first dose of the COVID vaccine [15,36,55,56]. It was suggested that a mixture of natural infection and vaccination stimulation led to a robust antibody response following 1D in individuals with SARS-CoV-2 infection history [57]. However, it was noted that after receiving the mRNA vaccine second dose, COVID-19-recovered persons did not develop a recall antibody response. A study showed that following 1D of mRNA-1273, persons who had previously been infected with SARS-CoV-2 exhibited a strong humoral response. Nevertheless, there was no rise in antibody titers following 2D [58].

In the current study, 42.0% of the individuals had not previously experienced COVID-19 symptoms, and there were anti-SARS-CoV-2 IgM- and IgG-positive cases among them, with a prevalence of 13.1% and 33.3%, respectively. Individuals who are asymptomatic carriers of SARS-CoV-2 may provide a risk for transmission. A study found that even asymptomatic persons may carry SARS-CoV-2 viral RNA after being exposed to COVID-19 cases, and the carriage appears to be long-lasting. The long duration of asymptomatic infection with SARS-CoV-2 may need a rethinking of quarantine periods during epidemics and pandemics. To prevent and control future SARS-CoV-2 infections in their early stages, those who have had close contact with a confirmed SARS-CoV-2 case should be regularly observed and evaluated to rule out infection, even if they have no symptoms. The quarantine of asymptomatic carriers and the identification of their contacts are critical components of these successful control measures of SARS-CoV-2 [59]. Another study looked at asymptomatic SARS-CoV-2 carriers’ infectivity by checking the cytopathic effects of SARS-CoV-2 on Vero cells in longitudinally collected asymptomatic carriers’ nasopharyngeal samples. They found that asymptomatic carriers can shed live viruses up to 15 days following the initial positive PCR test. These findings provide light on the significance of SARS-CoV-2 carriers as a source of transmission, emphasizing the importance of asymptomatic carrier isolation policies [60].

In the performed study, all participants had never performed testing for SARS-CoV-2-RNA by RT-PCR before the serology testing, and none of them had ever been hospitalized due to COVID-19-like symptoms. This could suggest that the people who were screened, whether or not they had received a vaccination, had a robust enough immune response against SARS-CoV-2 that they did not require hospitalization or even RT-PCR.

The anti-SARS-CoV-2 immunological response of nonsmokers, people who exercise regularly, and those who take vitamin supplements, eat a balanced diet, and use certain herbs is noteworthy. Among the vaccinated subjects, 96.6%, 25.0%, 31.9%, 45.7%, and 7.8% of the IgG-positive group, versus 97.2%, 60.6%, 64.2%, 52.3%, and 6.4% of the IgG-positive non-vaccinated group, were nonsmokers, exercisers, and those taking vitamin supplements, eating a balanced diet, and using herbs, respectively. Furthermore, 93.5%, 32.3%, 35.5%, 48.4%, and 6.5% of the IgM-positive vaccinated group, versus 100.0%, 63.6%, 81.8%, 45.5%, and 4.5% of the IgM-positive non-vaccinated participants, were nonsmokers, physical exercisers, vitamin supplement users, balanced eaters, and herbalists, respectively.

Smoking may increase SARS-CoV-2 infection susceptibility by ACE2 receptors upregulation, which are the primary receptors used by the SARS-CoV-2 to enter host mucosa to cause active infection. Current smokers have higher gene expression of *ACE2* genes than prior smokers and nonsmokers [61]. The lungs and immune system are weakened by tobacco smoking, which lowers one’s capacity to combat COVID-19 [62]. Furthermore, smoking significantly influenced COVID-19 severity by aggravating many comorbidities [63], and many increase side effects following COVID-19 vaccination [64].

Researchers have demonstrated the influence of a physically active lifestyle on immune system. Frequent moderate exercise strengthens the immune system, which may help prevent viral illnesses, limit or postpone immunological aging, and increase responses to vaccines [65,66].

Vitamins have been shown to have immunomodulatory, anti-inflammatory, and antioxidant properties the therapy of COVID-19. It has been demonstrated that vitamin consumption alleviates cytokine storms, reduces oxidative stress markers, and can reduce severity of COVID-19 by lowering pro-inflammatory cytokines, hence preventing organ failure [67]. The important biological role of Vitamin D in improving inflammation, innate and adaptive immune systems is clear. Vitamin D appears to be helpful if a patient has severe COVID-19, possibly by calming the cytokine storm [68,69]. A lack of vitamin D-mediated regulation of pro-inflammatory cytokines may aggravate the cytokine storm found in specific subgroups of individuals with severe COVID-19 [70]. Participants who received the Pfizer BioNTech vaccine may experience less side effects after vaccination and have higher levels of IgG if they take 600 IU of vitamin D3 supplements [71]. Vitamin B may control COVID-19 symptoms, treat SARS-CoV-2 infection, shorten hospital stays, and assist in development and maintenance of a healthy immune system [72].

Diets and dietary supplements try to supply the body with vitamins, trace elements, and minerals to improve immune system function and human health. Zinc, selenium, magnesium, Vitamins A, B, C, D, glutamine, resveratrol, beta-glucans, iron, omega-3 fatty acids, and probiotics may help the immune system function, with benefits before, during, and after infection with SARS-CoV-2 [73]. Along with strengthening immune system, adequate nutritional supplementation has been demonstrated to block viral entrance by disrupting the membrane fusion process that follows ACE2 docking. It has been extensively demonstrated that probiotic and prebiotic supplements significantly stimulate the immune system, which, in turn, improves vaccine efficacy and antibody production. Likewise, extracts from herbal mixtures or herbal-derived compounds provide a broad range of immunoactive chemicals capable of maintaining the antiviral response, and can inhibit viral entrance, hence averting SARS-CoV-2 infection [74]. A balanced diet that is rich in macronutrients, dietary proteins, omega-3 fatty acids, and probiotics may help protect against SARS-CoV-2 by reducing inflammation, oxidative stress, and cytokine storms while also strengthening the immune system, and increasing survival rates of COVID-19 cases [75].

For the prevention and co-treatment of COVID-19 and associated complications, several dietary supplements show promise. A plant-based diet, the Mediterranean diet (MD) emphasizes a lot of vegetables, fruits, grains, tree nuts, seeds, legumes, and olives. An increased moderate intake of seafood and fish (≥2 servings/week), moderate weekly consumption of poultry, dairy products, and eggs (2 servings/week), low red meat consumption (≤2 servings/week), and processed meat (once weekly) are all associated with olive oil. These eating habits, including vitamins and minerals, have immunomodulatory, antioxidant, and anti-inflammatory effects. Interestingly, MD adherence is negatively correlated with the risk of COVID-19 comorbidities including obesity, diabetes, sepsis, cardiovascular diseases, and cancer [73].

Eighty percent of individuals utilize herbs or plants for therapeutic purposes, according to WHO statistics. Because medicinal plants’ secondary metabolites can bind to viral proteins and enzymes and reduce their function, they are believed to be helpful in reducing viral infections [76]. Curcumin and piperine were the most utilized herbal supplements among the participants. When used as an adjuvant for treating COVID-19 cases, the oral curcumin and piperine may dramatically reduce morbidity and death. Curcumin may be a natural and safe option to prevent the thromboembolic events after COVID-19 [77].

In Italy, the lockdown’s psychological effects and restrictions caused by the COVID-19 pandemic itself resulted in a decline in healthy Italian lifestyles. Consumption of unhealthy foods increased in this regard, but physical activity and adherence to the MD decreased [78]. Therefore, even if there is a lockdown during an anticipated future epidemic or pandemic, we should prioritize healthy eating and lifestyle choices, along with increasing physical activity.

Our study’s limitations include not evaluating the role of SARS-CoV-2 variants and not assessing the amplitude and persistence of anti-SARS-CoV-2 Igs between vaccinated and non-vaccinated groups. Nevertheless, we think that our results are valuable and that they bring up significant issues that need to be explored in additional research on how an individual’s anthropometrics, relevant COVID-19 history, and lifestyle contribute to the development of their humoral immunity against SARS-CoV-2, either following infection or vaccination.

## 5. Conclusions and Recommendations

To the best of our knowledge, this study is the first to screen for the seroprevalence of anti-SARS-CoV-2 IgM/IgG in newcomer healthy non-obese students (of about 18 years old, both vaccinated and non-vaccinated) at Kafr Elsheikh University in Egypt, and could be helpful to save a great deal of money and prevent future deadly outbreaks, epidemics, or pandemics by building on the lessons learnt during the SARS-CoV-2 pandemic. The prevalence of anti-SARS-CoV-2 antibodies was 56.3% for IgG and 13.3% for IgM. There were no statistically significant relationships between gender, BMI, and IgM or IgG positivity in both the vaccinated and unvaccinated groups. We should plan measures for dealing with future epidemics or pandemics caused by emerging or re-emerging coronaviruses. Persons who are free from comorbidities, young, non-obese, non-smokers, engage in physical exercise, take vitamins, eat a balanced diet, and use certain immunostimulant herbal supplements all have a strong anti-SARS-CoV-2 humoral immune response, even if they were not vaccinated. During expected pandemics, the vaccination of this group should not be a priority to preserve vaccine doses for high-risk vulnerable people.

## Figures and Tables

**Table 1:** Comparison of participants’ vaccination status and anti-SARS-CoV-2 IgG/IgM positivity in their blood (total *n* = 400). The displayed data are frequencies; *n*. (%).

Antibodies Positivity	Total (*n* = 400)	Non-Vaccinated (*n* = 200)	Vaccinated (*n* = 200)	χ^2^	*p*
**IgG**	225 (56.3%)	109 (54.5%)	116 (58.0%)	0.498	0.480
**IgM**	53 (13.3%)	22 (11%)	31 (15.5%)	1.762	0.184
**IgG only**	196 (49%)	96 (48%)	100 (50%)	0.160	0.689
**IgM only**	24 (6%)	9 (4.5%)	15 (7.5%)	1.596	0.207
**IgG and IgM**	29 (7.3%)	13 (6.5%)	16 (8%)	0.335	0.563

χ^2^: chi square test. *p*: *p* value for comparison between the two studied groups. Statistical significance is at *p* ≤ 0.05.

**Table 2:** Comparison of participants’ vaccination type and anti-SARS-CoV-2 IgG/IgM positivity in their blood (total *n* = 400). The presented data are frequencies; *n*. (%).

Antibodies Positivity	Total (*n* = 400)	Non-Vaccinated (*n* = 200)	Vaccinated: Type of Vaccine			χ^2^	*p*
			Pfizer-BioNTech (*n* = 28)	Oxford-AstraZeneca (*n* = 51)	Sinopharm/Sinovac-CoronaVac (*n* = 121)		
**IgG**	225 (56.3%)	109 (54.5%)	12 (42.9%)	25 (49.0%)	79 (65.3%)	7.391	0.060
**IgM**	53 (13.3%)	22 (11%)	5 (17.9%)	7 (13.7%)	19 (15.7%)	2.041	0.564
**IgG only**	196 (49%)	96 (48%)	10 (35.7%)	21 (41.2%)	69 (57%)	6.425	0.093
**IgM only**	24 (6%)	9 (4.5%)	3 (10.7%)	3 (5.9%)	9 (7.4%)	2.770	^MC^*p* = 0.395
**IgG and IgM**	29 (7.3%)	13 (6.5%)	2 (7.1%)	4 (7.8%)	10 (8.3%)	0.603	^MC^*p* = 0.907

χ^2^: chi square test. MC: Monte Carlo test. *p*: *p* value for comparison between the two studied groups. Statistical significance is at *p* ≤ 0.05.

**Table 3:** Comparison of participants’ number of vaccination doses and anti-SARS-CoV-2 IgG/IgM positivity in their blood (total *n* = 400). The data shown are frequencies; *n*. (%).

Antibodies Positivity	Total (*n* = 400)	Non-Vaccinated (*n* = 200)	Vaccinated: Number of Doses		χ^2^	*p*
			One Dose (*n* = 8)	Two Doses (*n* = 192)		
**IgG**	225 (56.3%)	109 (54.5%)	8 (100%)	108 (56.3%)	6.950 *	^MC^*p* = 0.029 *
**IgM**	53 (13.3%)	22 (11%)	2 (25%)	29 (15.1%)	2.416	0.299
**IgG only**	196 (49%)	96 (48%)	6 (75%)	94 (49%)	2.141	^MC^*p* = 0.365
**IgM only**	24 (6%)	9 (4.5%)	0 (0%	15 (7.8%)	2.427	0.297
**IgG and IgM**	29 (7.3%)	13 (6.5%)	2 (25%)	14 (7.3%)	3.916	0.141

χ^2^: chi square test. MC: Monte Carlo test. *p*: *p* value for comparison between the two studied groups. *: Statistically significant at *p* ≤ 0.05.

**Table 4:** Comparison of vaccinated participants’ anthropometrics, COVID-19 history, and lifestyle versus anti-SARS-CoV-2 IgM/IgG negativity or positivity in their blood (total *n* = 200). Frequencies are displayed as *n*. (%) and mean ± SD (median; range).

		Total (*n* = 200)	Anti-SARS-CoV-2 IgM		Test of Sig.	*p*_1_	Anti-SARS-CoV-2 IgG		Test of Sig.	*p*_2_
			Negative (*n* = 169)	Positive (*n* = 31)			Negative (*n* = 84)	Positive (*n* = 116)		
**Gender**	Male	83 (41.5%)	69 (40.8%)	14 (45.2%)	χ^2^ = 0.203	0.653	38 (45.2%)	45 (38.8%)	χ^2^ = 0.834	0.361
	Female	117 (58.5%)	100 (59.2%)	17 (54.7%)			46 (54.8%)	71 (61.2%)		
**Age (Years)**	Mean ± SD.	18.1 ± 0.3	18.1 ± 0.3	18.2 ± 0.4	t = 0.662	0.509	18.2 ± 0.4	18.1 ± 0.3	t = 2.267 *	0.025 *
	Median (Min.–Max.)	18.0 (18.0–19.0)	18 (18–19)	18 (18–19)			18.0 (18.0–19.0)	18.0 (18.0–19.0)		
**BMI (kg/m^2^)**	Mean ± SD.	25.3 ± 2.8	25.4 ± 2.8	24.9 ± 2.7	U = 2152.0	0.114	25.7 ± 3.3	25.1 ± 2.4	U = 4410.50	0.253
	Median (Min.–Max.)	25.7 (18.4–35.4)	25.8 (18.4–35.4)	24.7 (20.5–35.4)			25.9 (19.5–35.4)	25.7 (18.4–31.6)		
**IgM**	Negative	169 (84.5%)					69 (82.1%)	100 (86.2%)	χ^2^ = 0.614	^FE^*p* = 0.433
	Positive	31 (15.5%)					15 (17.9%)	16 (13.8%)		
**IgG**	Negative	84 (42.0%)	69 (40.8%)	15 (48.4%)	χ^2^ = 0.614	0.433				
	Positive	116 (58.0%)	100 (59.2%)	16 (51.6%)						
**Q1** **—** **Previous COVID-19-like symptoms**	No	92 (46%)	77 (45.6%)	15 (48.4%)	χ^2^ = 0.084	0.772	62 (73.8%)	30 (25.9%)	χ^2^ = 45.091 *	<0.001 *
	Yes	108 (54%)	92 (54.4%)	16 (51.6%)			22 (26.2%)	86 (74.1)		
**Q1** **—** **Previous hospital isolation due to COVID-19-like symptoms (*n* = 108)**	No	108 (54%)	92 (54.4%)	16 (51.6%)	–	–	22 (26.2%)	86 (74.1)	–	–
	Yes	0 (0%)	0 (0%)	0 (0%)			0 (0%)	0 (0%)		
**Q2** **—** **Previous RT-PCR-positive SARS-CoV-2 diagnosis**	No	200 (100%)	169 (100%)	31 (100)	–	–	84 (100%)	116 (100)	–	–
	Yes	0 (0%)	0 (0%)	0 (0%)			0 (0%)	0 (0%)		
**Q3** **—** **Previous RT-PCR-negative SARS-CoV-2 test**	No	200 (100%)	169 (100%)	31 (100)	–	–	84 (100%)	116 (100)	–	–
	Yes	0 (0%)	0 (0%)	0 (0%)			0 (0%)	0 (0%)		
**Q4** **—** **Contact with a person having a positive SARS-CoV-2-RNA PCR test**	No	167 (83.5%)	141 (83.4%)	26 (83.9%)	χ^2^ = 0.004	0.952	66 (78.6%)	101 (87.1%)	χ^2^ = 2.553	0.110
	Yes	33 (16.5%)	28 (16.6%)	5 (16.1%)			18 (21.4%)	15 (12.9%)		
**Q5** **—** **Contact with a person suffering from COVID-19-like symptoms without RT-PCR testing or with negative RT-PCR results**	No	80 (40%)	69 (40.8%)	11 (35.5%)	χ^2^ = 0.312	0.577	37 (44%)	43 (37.1%)	χ^2^ = 0.989	0.320
	Yes	120 (60%)	100 (59.2%)	20 (64.5%)			47 (56%)	73 (62.9%)		
**Smoking**	No	188 (94.0%)	159 (94.1%)	29 (93.5%)	χ^2^ = 0.013	^FE^*p* = 1.000	76 (90.5%)	112 (96.6%)	χ^2^ = 3.189	0.074
	Yes	12 (6.0%)	10 (5.9%)	2 (6.5%)			8 (9.5%)	4 (3.4%)		
**Physical activity**	No	153 (76.5%)	132 (78.1%)	21 (67.7%)	χ^2^ = 1.565	0.211	66 (78.6%)	87 (75.0%)	χ^2^ = 0.346	0.557
	Yes	47 (23.5%)	37 (21.9%)	10 (32.3%)			18 (21.4%)	29 (25.0%)		
**Vitamin supplements**	No	145 (72.5%)	125 (74.0%)	20 (64.5%)	χ^2^ = 1.173	0.279	66 (78.6%)	79 (68.1%)	χ^2^ = 2.678	0.102
	Yes	55 (27.5%)	44 (26.0%)	11 (35.5%)			18 (21.4%)	37 (31.9%)		
**Healthy diet**	No	118 (59.0%)	102 (60.4%)	16 (51.6%)	χ^2^ = 0.828	0.363	55 (65.5%)	63 (54.3%)	χ^2^ = 2.511	0.113
	Yes	82 (41.0%)	67 (39.6%)	15 (48.4%)			29 (34.5%)	53 (45.7%)		
**Herbal supplements**	No	184 (92.0%)	155 (91.7%)	29 (93.5%)	χ^2^ = 0.120	1.000	77 (91.7%)	107 (92.2%)	χ^2^ = 0.022	0.882
	Yes	16 (8.0%)	14 (8.3%)	2 (6.5%)			7 (8.3%)	9 (7.8%)		

SD: standard deviation. t: Student’s *t*-test. U: Mann–Whitney test. χ^2^: chi-square test. FE: Fisher’s exact test. *p*_1_: *p* value for comparing between negative and positive in anti-SARS-CoV-2 IgM. *p*_2_: *p* value for comparing between negative and positive in anti-SARS-CoV-2 IgG. *: Statistically significant at *p* ≤ 0.05.

**Table 5:** Comparison of non-vaccinated participants’ anthropometrics, COVID-19 history, and lifestyle versus anti-SARS-CoV-2 IgM/IgG negativity or positivity in their blood (total *n* = 200). Frequencies are presented as *n*. (%) and mean ± SD (median; range).

		Total (*n* = 200)	Anti-SARS-CoV-2 IgM		Test of Sig.	*p*_1_	Anti-SARS-CoV-2 IgG		Test of Sig.	*p*_2_
			Negative (*n* = 178)	Positive (*n* = 22)			Negative (*n* = 91)	Positive (*n* = 109)		
**Gender**	Male	90 (45.0)	76 (42.7%)	14 (63.6%)	χ^2^ = 3.469	0.063	43 (47.3%)	47 (43.1%)	χ^2^ = 0.342	0.558
	Female	110 (55.0)	102 (57.3%)	8 (36.4%)			48 (52.7%)	62 (56.9%)		
**Age (Years)**	Mean ± SD.	18.2 ± 0.4	18.15 ± 0.35	18.2 ± 0.4	t = 0.441	0.660	18.2 ± 0.4	18.1 ± 0.3	t = 1.697	0.091
	Median (Min.–Max.)	18.0 (18.0–19.0)	18.0 (18.0–19.0)	18.0 (18.0–19.0)			18.0 (18.0–19.0)	18.0 (18.0–19.0)		
**BMI (kg/m^2^)**	Mean ± SD.	25.35 ± 2.58	25.3 ± 2.7	25.6 ± 1.9	U = 1855.50	0.689	25.5 ± 2.7	25.2 ± 2.5	U = 4618.000	0.402
	Median (Min.–Max.)	25.7 (18.4–35.4)	25.7 (18.4–35.4)	25.6 (21.8–29.4)			25.8 (19.5–35.4)	25.7 (18.4–35.4)		
**IgM**	Negative	178 (89.0)					82 (90.1%)	96 (88.1%)	χ^2^ = 0.210	0.647
	Positive	22 (11.0)					9 (9.9%)	13 (11.9%)		
**IgG**	Negative	91 (45.5)	82 (46.1%)	9 (40.9%)	χ^2^ = 0.210	0.647				
	Positive	109 (54.5)	96 (53.9%)	13 (59.1%)						
**Q1** **—** **Previous COVID-19-like symptoms**	No	76 (38%)	69 (38.8%)	7 (31.8%)	χ^2^ = 0.401	0.527	50 (54.9%)	26 (23.9%)	χ^2^ = 20.350 *	<0.001 *
	Yes	124 (62%)	109 (61.2%)	15 (68.2%)			41 (45.1%)	83 (76.1%)		
**Q1** **—** **Previous hospital isolation due to COVID-19-like symptoms (*n* = 124)**	No	124 (62%)	109 (61.2%)	15 (68.2%)	–	–	41 (45.1%)	83 (76.1%)	–	–
	Yes	0 (0%)	0 (0%)	0 (0%)			0 (0%)	0 (0%)		
**Q2** **—** **Previous RT-PCR-positive SARS-CoV-2 diagnosis**	No	200 (100%)	178 (100%)	22 (100%)	–	–	91 (100%)	109 (100%)	–	–
	Yes	0 (0%)	0 (0%)	0 (0%)			0 (0%)	0 (0%)		
**Q3** **—** **Previous RT-PCR-negative SARS-CoV-2 test**	No	200 (100%)	178 (100%)	22 (100%)	–	–	91 (100%)	109 (100%)	–	–
	Yes	0 (0%)	0 (0%)	0 (0%)			0 (0%)	0 (0%)		
**Q4** **—** **Contact with a person having a positive SARS-CoV-2-RNA PCR test**	No	148 (74%)	135 (75.8%)	13 (59.1%)	χ^2^ = 2.856	0.091	63 (69.2%)	85 (78%)	χ^2^ = 1.974	0.160
	Yes	52 (26%)	43 (24.2%)	9 (40.9%)			28 (30.8%)	24 (22%)		
**Q5** **—** **Contact with a person suffering from COVID-19-like symptoms without RT-PCR test or with negative RT-PCR results**	No	70 (35%)	64 (36%)	6 (27.3%)	χ^2^ = 0.649	0.421	33 (36.3%)	37 (33.9%)	χ^2^ = 0.117	0.732
	Yes	130 (65%)	114 (64%)	16 (72.7%)			58 (63.7%)	72 (66.1%)		
**Smoking**	No	193 (96.5%)	171 (96.1%)	22 (100.0%)	χ^2^ = 0.897	^FE^*p* = 1.000	87 (95.6%)	106 (97.2%)	χ^2^ = 0.397	^FE^*p* = 0.704
	Yes	7 (3.5%)	7 (3.9%)	0 (0.0%)			4 (4.4%)	3 (2.8%)		
**Physical activity**	No	75 (37.5%)	67 (37.6%)	8 (36.4%)	χ^2^ = 0.014	0.907	32 (35.2%)	43 (39.4%)	χ^2^ = 0.388	0.533
	Yes	125 (62.5%)	111 (62.4%)	14 (63.6%)			59 (64.8%)	66 (60.6%)		
**Vitamin supplements**	No	71 (35.5%)	67 (37.6%)	4 (18.2%)	χ^2^ = 3.238	0.072	32 (35.2%)	39 (35.8%)	χ^2^ = 0.008	0.928
	Yes	129 (64.5%)	111 (62.4%)	18 (81.8%)			59 (64.8%)	70 (64.2%)		
**Healthy diet**	No	97 (48.5%)	85 (47.8%)	12 (54.5%)	χ^2^ = 0.362	0.548	45 (49.5%)	52 (47.7%)	χ^2^ = 0.060	0.806
	Yes	103 (51.5%)	93 (52.2%)	10 (45.5%)			46 (50.5%)	57 (52.3%)		
**Herbal supplements**	No	169 (84.5%)	148 (83.1%)	21 (95.5%)	χ^2^ = 2.265	^FE^*p* = 0.210	67 (73.6%)	102 (93.6%)	χ^2^ = 15.073 *	<0.001 *
	Yes	31 (15.5%)	30 (16.9%)	1 (4.5%)			24 (26.4%)	7 (6.4%)		

SD: standard deviation. t: Student’s *t*-test. U: Mann–Whitney test. χ^2^: chi-square test. FE: Fisher’s exact test. *p*_1_: *p* value for comparing between negative and positive in anti-SARS-CoV-2 IgM. *p*_2_: *p* value for comparing between negative and positive in anti-SARS-CoV-2 IgG. *: Statistically significant at *p* ≤ 0.05.

## Data Availability

All data are available in the manuscript.
